# Phytochemical characterization, antimicrobial and antioxidant activities of *Terminalia catappa* methanol and aqueous extracts

**DOI:** 10.1186/s12906-024-04449-7

**Published:** 2024-04-02

**Authors:** Wangui Clement Mwangi, Walyambillah Waudo, Madivoli Edwin Shigwenya, Joyline Gichuki

**Affiliations:** 1 Chemistry Department, Jomo Kenyatta University of Agriculture and Technology, P.O Box 62000-00200, Nairobi, Kenya; 2 Pharmaceutical Chemistry Department, Mount Kenya University, P.O. Box 342-01000, Thika, Kenya

**Keywords:** *T. catappa* L., Antimicrobial activity, Antioxidant activity

## Abstract

**Background:**

A study carried out by World Health Organization revealed that around 80% of individuals globally depends on herbal forms of medication with 40% of pharmaceutical products being sourced from medicinal plants. The study objective was to evaluate the phytochemicals composition, in vitro antimicrobial and antioxidant properties of the leaves of Terminalia catappa L. aqueous and methanolic extracts.

**Methods:**

Antimicrobial activity was analyzed by disk diffusion, the minimum inhibitory concentration in-vitro assays with ciprofloxacin as the standard for antibacterial assay while nystatin for antifungal assay. Ferric reducing antioxidant power and 2,2-diphenyl-1-picryl-hydrazyl-hydrate assays were used for the evaluation of antioxidant properties of the crude extracts while the groups responsible for this activity identified using Fourier transform infrared spectrophotometer.

**Results:**

The study found that the leaves of Terminalia catappa contained alkaloids, tannins, steroids, cardiac glycosides, flavonoids, phenols, saponins, and coumarins, but terpenoids were absent. Presence of functional groups associated with this class of compounds such as OH vibrational frequencies were observed in IR spectrum of the crude extracts. Methanolic extract from Terminalia catappa exhibited greater antibacterial properties against Pseudomonas aeruginosa, Escherichia coli and Staphylococcus aureus, whereas aqueous extract displayed greater antibacterial activity against Bacillus subtilis for all concentrations tested. The amount of the sample that scavenged 50 percent of DPPH (IC50) was found to be 8.723, 13.42 and 13.04 µg/mL for L-ascorbic acid, Terminalia catappa L. methanolic and aqueous extracts respectively. The antimicrobial and antioxidant activities varied with the extract concentration and solvent used in extractions.

**Conclusion:**

*Terminalia catappa* L. leaves are prospective for use as a source of therapeutic agents that could lead to the advancement of new antimicrobial and antioxidant products.

**Supplementary Information:**

The online version contains supplementary material available at 10.1186/s12906-024-04449-7.

## Introduction

Herbal medicine has been acknowledged as a reservoir of bioactive phytochemicals that can be employed to address and avert diverse human health predicaments, whether chronic or acute. The manifestation of its ethno-pharmacological advantages has resulted in approximately 80% of the population in developing nations relying on herbal medicine as their primary source of healthcare [1]. Additionally, herbal medicine has been comprehensively incorporated as a component of complementary and alternative medicine due to its effectiveness, safety, and cost-effectiveness [2]. Biologically active compounds vary greatly from plant to plant, soil to soil, and from micro-organism to micro-organism [3].

Therapeutic plants, in addition to essential metabolites, also contain secondary metabolites such as flavonoids, alkaloids, phenolic compounds, steroids, glycosides, and tannins. These compounds are extremely important alternative therapies for wound healing, whether individually or in combination. Herbal plants are easily affordable and accessible to everyone, especially in the third world, and can be used as antioxidants against free radicals which cause many diseases in humans [4]. Antimicrobials play an essential role in curbing the prevalence of infectious diseases around the world. However, the spread of multi-drug resistant (MDR) bacteria has become a major public health concern. This is because there are a handful or even no effective antimicrobials to treat an infection caused by resistant pathogenic bacterium. Therefore, with the global spread of drug-resistant clinical isolates on the rise, the search for new antimicrobials is urgent [5].

The *T. catappa* L. (Combretaceae) commonly referred to as tropical almond or Indian almond is traditionally used as an anti-inflammatory, antimicrobial, hepatoprotective, vermifuge, antioxidant, anticancer, and antidiabetic agent [2]. A study on the *T. catappa L.* leaves methanol extract showed 70.4% ± 4.9 DPPH scavenging activity and 99% ± 1.6 for the same concentration of extract and L-ascorbic acid respectively. According to the study, the *T. catappa L.* methanol extract had an IC_50_ of 5.6 µg/ml for DPPH scavenging activity. The *T. catappa* L. also contained scavenging activity for reactive oxygen species such as hydroxyl, superoxide, and peroxide radicals [6]. Studies have shown that the leaves and barks of the plant exhibit antioxidant, hepato-protective, anti-inflammatory, antidepressant, antifungal, and chemopreventive activities. As a constituent in topical applications for wound healing, it has previously been reported that the *T. catappa* L. extracts exhibited reduction in wound healing properties in rats by 97% compared to betadine ointment. The ethanol extract of the *T. catappa L.* leaves displayed anti-inflammatory activity in animal models [6].

This study therefore sought to carry out phytochemical characterization of the crude extracts using a Fourier transform infrared spectrophotometer (FTIR). The study also sought to evaluate the antimicrobial properties of the *T.catappa L.* extracts by disc diffusion assay, and the antioxidant properties of the crude extracts by Free radical scavenging activity using DPPH and Potassium ferric reducing power (pFRAP) respectively.

## Methods

### Collection of plant materials

The *T. catappa L.* material was collected from Msambweni sub-county, Kwale County in Kenya, and transported to Mount Kenya University Pharmacognosy laboratory. The plant materials were authenticated at the Department of East African Herbarium, in the Museum of Kenya by Mr. Kennedy Matheka and given the reference number NMK/BOT/CTX/2/2ID/2023. A voucher specimen was deposited at The East African herbarium department, Museum Kenya. The plant materials were washed in running water and then rinsed off with deionized water. They were then air-dried in the shade for 4 weeks. After drying, the materials were grounded into a medium-fine powder using the laboratory grinder. [7].

### Extraction of samples

#### Methanol extraction

Approximately 200 g of the dried powder was subjected to cold maceration using 600 ml of methanol for extraction. The solution was placed on a mechanical shaker to facilitate continuous shaking for 72 h [8, 9]. The mixture was then filtered by 125 mm Whatman filter papers 1 and concentrated by use of a rotary vacuum evaporator, Stuart RE300DB (Bibby Scientific Limited, UK). at 55℃. The concentrated extract was stored in a pre-weighed reagent bottle and placed in an oven at 35℃. The process was repeated two consecutive times to obtain an adequate amount of the extract. After drying the extract was weighed and then refrigerated at 4℃ for future use [10].

#### Aqueous extraction

Approximately 200 g of the dried material was extracted using 600 ml of de-ionized water by heating at 60℃ for one hour in a water bath (Stuart SWB series). The solution was allowed to cool and filtered using 125 mm Whatman filter paper 1. The filtrate was transferred into a lyophilizer flask and dried by lyophilization for two days. The dried plant material was stored in a clean, dry, and labeled reagent bottle and stored in a refrigerator at 4℃ [10].

### Phytochemical screening

The screening of phytochemical constituents was done to determine the existence or non-existence of the phytochemicals [11]. This was done by visual inspection of colour changes or precipitation [12, 13].

#### Test for tannins

Approximately 5mg of the plant powder was weighed and put in a test tube. A few drops of FeCl_3_ and 10ml of distilled water were added and the solution boiled in a water bath. The presence of a blue-black precipitate indicated the presence of tannins in the test samples [14].

#### Test for alkaloids

For the test of alkaloids 5mg plant powder was taken and dissolved in 10ml of methanol. The solution was boiled in a water bath and filtered using a Whatman filter paper 1. Exactly 1 ml of 1% HCl was added, and then 6 drops of Dragendorff reagent was added to the mixture. The presence of alkaloids was indicated by the formation of a brownish-red precipitate [14].

#### Test for saponins

Approximately 5mg of the powder was weighed, transferred into a test tube, and dissolved in 5 ml of distilled water. The solution was filtered and 0.5ml of the filtrate was diluted to 5ml with distilled water. After vigorous shaking of the solution for 2 min, a stable foam formed showing the existence of saponins [14].

#### Test for flavonoids

To 3ml of the aqueous filtrate 3ml of dilute ammonia solution was added, followed by concentrated sulphuric acid. The presence of flavonoids was indicated by yellow colouration [14].

#### Test for steroids

For the test of steroids, 10 ml of distilled water was added to 5mg of the plant powder. The solution was filtered and to 1ml of the filtrate 1ml of chloroform and 1.5ml of concentrated sulphuric acid were added. The formation of a reddish-brown colour at the interface indicated the presence of steroids [15].

#### Test for coumarins

Approximately 1 ml of the aqueous filtrate was added to 1 ml of 10% sodium hydroxide solution. The appearance of a yellow colour indicated the presence of coumarins [15].

#### Test for terpenoids

Approximately 5mg of the plant powder was dissolved in 10 ml of chloroform. The solution was filtered and 3ml of concentrated sulphuric acid was added to 2ml of the filtrate. The presence of terpenoids was depicted by the reddish-brown ring formed at the interface [16].

#### Test for cardiac glycosides

The presence of cardiac glycosides was determined by taking 2ml of aqueous filtrate and reacting it with 1ml of glacial acetic acid containing a few drops of FeCl_3_. Exactly 6 drops of concentrated sulphuric acid were then added to the solution. The green–blue colour formed showed cardiac glycosides were present [15].

#### Test for phenols

Approximately 5mg of the plant material was dissolved in 10 ml of absolute ethanol and boiled for 10 min. The solution was filtered after cooling and a few drops of ferric chloride was added to the filtrate. The phenol's presence was shown by a green precipitate formed at the interface [17].

### Determination of functional group present in the extracts

Spectroscopic data was collected using an Affinity -1S FTIR infrared spectrophotometer Shimadzu Corp., 03191 (Shimadzu Corporation, Japan) equipped with attenuated total reflection (ATR). The instrument was configured to perform a total of 20 scans with a spectral resolution of 4 cm^−1^ for the background and sample spectra, recorded rapidly in the range from 4000 to 400 cm^−1^. The ATR was cleaned with a wet piece of cotton wool and the crystal was examined spectrally to ensure no residue was retained from the preceding analysis. The measurements were done at room temperature by placing 2 mg of the sample on an ATR crystal. The acquired bands were analyzed and assigned functional groups by comparing them with the literature values [9, 18].

### Antimicrobial activity

The media, distilled water, and glassware were sterilized using a Memmert autoclave at 121 ℃ for 15 min. All the work related to the use of microorganisms was done in a Bio-flow Laminar cabinet. [19, 20].

#### Collection of culture microorganisms

The extract's antimicrobial susceptibility test was done by disc diffusion assay using four bacteria strains and a fungus. The reference microorganism species; American type cell culture (ATCC) of *Escherichia coli* ATCC 25,922, *Bacillus subtilis* ATCC 6051***,**** Pseudomonas aeruginosa* ATTC 27855, *Staphylococcus aureus* ATCC 25923*,* bacteria strains and *Candida albicans* ATTC 1023, were obtained from Department of Microbiology, Mount Kenya University. All the laboratory works were performed following the guidelines from the Clinical and Laboratory Standards Institute (CLSI) [7, 10, 21, 22].

#### Preparation of media and Inocula

Sabouraud dextrose agar (SDA) and Mueller Hinton agar (MHA) media were prepared following the manufacturer’s instructions. The media were sterilized in an autoclave, partially cooled to 50 ℃, and 20 ml transferred to sterile disposable plates to obtain a uniform depth. Bacterial cultures were sub-cultured in the MHA and fungus was sub-cultured in SDA media to obtain a young growing culture. The standard inoculum suspensions were prepared at 0.5 McFarland equivalent turbidity and adjusted to give a turbidity of 1 × 10^8^ cells or spores/ml by checking the OD at 600nm [10].

#### Preparation of the samples and standard

Three different concentrations, that is 100, 50, and 25 mg/ml, of the samples, were prepared by weighing 100mg of each extract using analytical balance and serial diluting with 5% Dimethyl sulfoxide (DMSO). The samples were then diluted by 5% of DMSO to obtain 50 mg/ml and 25/ml of the extracts. Standard stock solutions of ciprofloxacin and nystatin were prepared by separately weighing 10 mg of each and dissolving them with 10 ml of 5% DMSO. The standard stock solutions were diluted with 5% DMSO to obtain working standards of 0.15 mg/ml and 0.2 mg/ml of ciprofloxacin and nystatin respectively [5].

### Antimicrobial assay

#### Antibacterial assay

The test was carried out by use of 2-Gram negative and 2-Gram positive bacteria strains. Approximately 20 ml of the sterile media was poured carefully into each Petri dish, to produce a plate with uniform thickness of 4 mm. The media in the petri dishes was allowed to cool, producing a firm gel for plating [23]. After solidification of the media, the plates were inoculated by rubbing sterile cotton swabs that were dipped into bacterial suspensions over the entire surface of the plate [24].

Sterile paper disks (6 mm in diameter), were used to aid in diffusion of the extract in the inoculated media in the plates. The test extracts together with negative and positive controls were applied to the disks by use of a fixed micro pipette set to deliver 10 μl. The positive control contained 0.15 mg/ml of ciprofloxacin while the negative control contained 5% DMSO. Each of the applications was done in triplicates and a pre-diffusion period of 30 min was allowed to facilitate diffusion before being placed in the incubator for 24 h at 37 ℃. Following incubation, the zones of inhibition around the disks correspond to the antibacterial activities of the tested extracts and were measured in mm. [5].

#### Antifungal assay

The antifungal activity was carried out with *Candida albicans* fungus on Sabouraud Dextrose agar media. The test was carried out by disk diffusion method with nystatin as the positive control. The sterilized media was poured onto the plastic plate and allowed to solidify. The samples, positive and negative controls were applied to the inoculated media, in triplicates. After pre-diffusion of the samples for 30 min, they were incubated for 24 h at 37 ℃. The inhibited zones were measured and recorded in mm after completion of the incubation period [24].

#### Determination of Minimum inhibition concentration (MIC)

The Broth micro-dilution assay was used to evaluate the MICs of the extract against the test microorganism. The MIC was determined only for the extracts that showed some zones of inhibition in the susceptibility study [10]. Exactly 25mg of each extract was weighed and diluted in 1 ml of 5% DMSO. The positive control was made by dissolving 5 mg of ciprofloxacin in 2 ml of 5% DMSO followed by serial dilution to obtain a concentration of 2 μg/ml ciprofloxacin. The Muller Hinton broth was prepared as per the manufacturer’s instructions and autoclaved for 15 min at 121℃. Exactly 100 μl of the sterile media was measured and transferred into a sterile 96-well plate. The extracts, positive and negative control were added to the wells media, and two-fold serial dilutions of the test solutions was done using a micropipette [25]. The inoculum was added to the wells containing the test samples and controls. The plates were covered with a sterile cover and incubated for 24 h at 37 ℃. After the incubation period, 100 μl of 0.34 mg/ml resazurin dye was added to each well and then re-incubated for 30 min [26]. The dye changed from a blue colour of resazurin to a pink colour of resofurin indicating bacteria growth in wells where the test sample was not able to inhibit the growth [27].

The minimum bactericidal (MBC) and fungicidal (MFC) concentrations were determined by directly plating the well contents at a concentration greater than the MIC. The value of MBC or MFC was evaluated by determining the concentration of the well where the microorganism did not grow after 24 h of incubation at 37 ℃ [25].

### Antioxidant assay

#### Determination of antioxidant activity

The antioxidant assay of the *T. catappa* L. extract was determined using the free radical scavenging properties of DPPH. To measure the property, different concentrations of test samples dissolved in methanol were prepared from 1000 µg/ml to 0.32 µg/ml by serial dilution method. The L-ascorbic acid and methanol were prepared the same way as the sample to act as the control. For the measurement of absorbance of the test solutions by UV–Vis spectrophotometer, Microprocessor double beam model: AVI-2704 (Avi scientific India) 517 nm was used, with methanol as the blank. The radical scavenging activities (RSA) of the studied extracts were calculated using the formula below [28].

$$\%\text{RSA}=\frac{\text{Abs}.\;\mathrm{of}\;\mathrm{DPPH}\;\mathrm{solution}-\text{Abs}.\mathrm{of}\;\mathrm{the}\;\mathrm{test}\;\mathrm{sample}}{\text{Abs}.\;\mathrm{of}\;\mathrm{DPPH}\;\mathrm{solution}}\text{x}100$$

The amount at which the sample gives 50% inhibition (IC50) scavenging activity was determined by plotting the graph against the concentrations of the sample.

#### Determination of potassium ferricyanide reducing antioxidant power (pFRAP)

The ferricyanide-reducing antioxidant activity of the methanol and aqueous extracts was determined using [29], method with minor modifications. Potential antioxidant interacts with a colourless Fe^3+^ complex to reduce it into intense blue Fe^2+^, which is the basis of the ferric-reducing antioxidant power assay (FRAP) [30].

Exactly 1 ml of the test solutions or L-Ascorbic acid was combined with 2.5 ml of 0.03 M potassium ferricyanide and 2.5 ml of phosphate buffer (0.2 M, pH 6.6). The concentrations of the extract solution or L-Ascorbic acid ranged from 0.32 μg/ml to 1000 μg/ml. The mixtures were then incubated in a water bath at 50 ℃ for 20 min. After cooling 2.5 ml of 0.6M Trichloroacetic Acid (TCA) was added to each of the test solutions. To an aliquot of 2 ml of each solution, 0.5 ml of FeCl_3_ solution (0.01%) and 2 ml of distilled water were added. The absorbance of the test solutions was measured at 700 nm by a double-beam Uv Vis spectrophotometer [29].

## Results

### Percentage yield

Following the extraction, the percentage yield of each extract was determined. The methanol extract gave a yield of 7.1% while aqueous extract gave 18.3%.

### Screening of phytochemical constituents

A qualitative analysis of the *T. catappa* L. methanol and aqueous extracts was conducted to evaluate the presence and absence of flavonoids, saponins alkaloids, tannins, terpenoids, steroids, coumarins, phenols, and cardiac glycosides. Both methanol and aqueous extracts exhibited various phytochemical compounds as outlined in Table 1.

**Table 1:** Phytochemical screening of T. catappa L. methanol and aqueous extracts

Phytochemical compounds	Extracts	
	**Methanol**	**Aqueous**
Tannins	** + **	** + **
Saponins	** + **	** + **
Phenols	** + **	** + **
Alkaloids	** + **	** + **
Flavonoids	** + **	** + **
Terpenoids	** − **	** − **
Steroids	** + **	** + **
Cardiac glycosides	** + **	** + **
Coumarins	** + **	** + **

**Key: +  = **Presence—= absence

### Determination of functional groups of the extract by FTIR

**Fig. 1:**
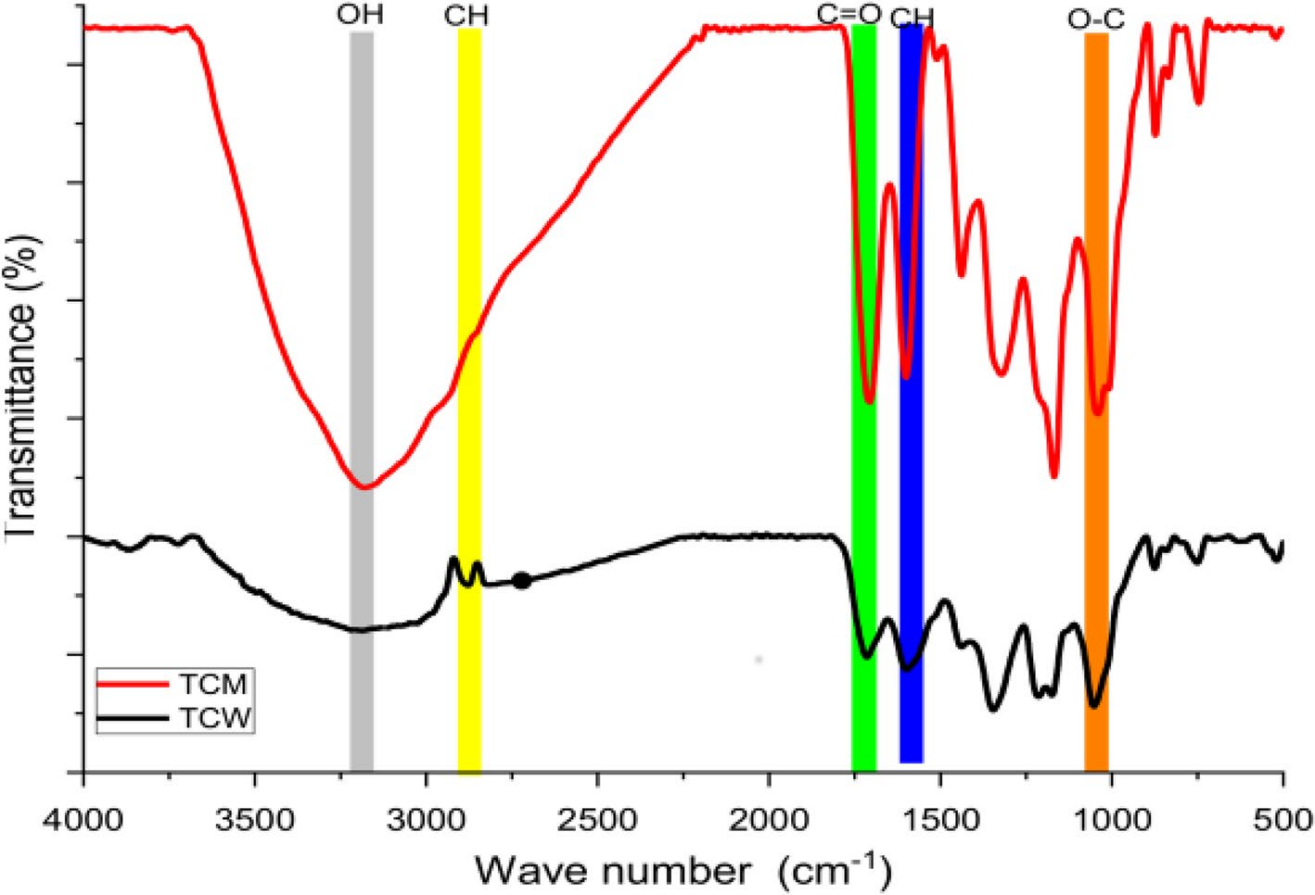
IR spectrum of the *T. catappa* L. methanol (TCM) and aqueous extracts (TCW)

Based on the absorption band values, FTIR was used to identify the functional groups of the active compounds in the *T. catappa* L. aqueous and methanol extracts, and the results are depicted in Fig. 1. The IR spectra of the *T. catappa* L. methanolic and aqueous extracts had a broad band at 3171 cm^−1^ which is distinctive of O–H stretching [2]. This can be linked to different compounds that bear the OH vibrational groups such as saponins and carboxylic acids [31]. The absorption bands at 1712 cm^−1^ and 1596 cm^−1^ are distinctive of carbonyl (C = O) and C = C stretching respectively. The band at a frequency of 1322 cm^−1^ is attributed to C-H bending while the other notable absorption bands at 1041 and 1165 cm^−1^ were due to C-O stretching indicating the presence of oxygen-containing groups such as phenols, anhydrides, or alcohols [31].

### Antimicrobial activity

**Fig. 2:**
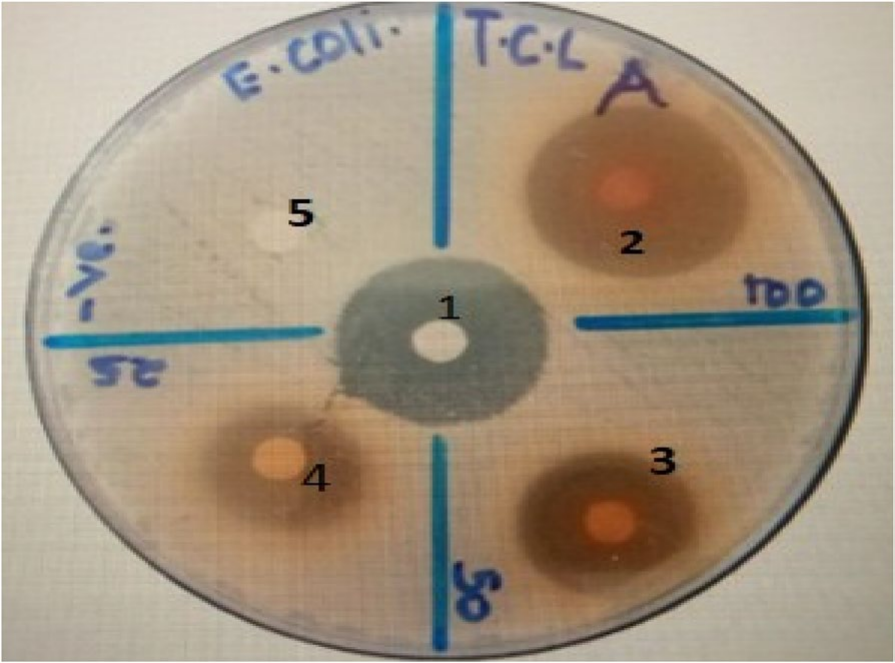
Zones of inhibition of ciprofloxacin (1), 5%DMSO (5), and the *T. catappa* L. methanol extract at 100 *mg/ml* (2), 50 *mg/ml* (3), and 25 mg/ml (4) against *E. coli* bacteria

In this study, both methanol and aqueous extracts of the *T. catappa* L. extracts produced significant zones of inhibition against the tested bacteria strains as depicted in Fig. 2 and Table 2. Measurements in the following ranges were used to determine the bacterial culture's susceptibility to extract: 0 to 7 mm denotes inactivity, 8 to 12 mm, weak activity, and 12 mm and above, strong activity [32].

**Table 2:** Table showing the average diameter of zones of inhibition of the T. catappa L. methanol (TCM) and aqueous (TCW) extracts against five microorganisms

	**Mean ± SD**				
Sample	***S. aureus***	***P. aeruginosa***	***E. coli***	***B. subtilis***	***C. albicans***
0.15 mg/ml ciprofloxacin	21.0 ± 0^Aa^	27.7 ± 0.33^Ab^	25.7 ± 0.17^Ac^	26.3 ± 0.33^Ad^	
100 mg/ml TCM	18.5 ± 0.29^Ba^	25.7 ± 0.33^Bb^	25.0 ± 0.0^Ac^	14.3 ± 0.33^Bd^	20.3 ± 0.58^Ae^
100 mg/ml TCW	15.3 ± 0.33^Ca^	15.7 ± 0.33^Ca^	20.3 ± 0.33^Bb^	19.3 ± 0.33^Cb^	18.3 ± 0.58^Bc^
50 mg/ml TCM	13.7 ± 0.58^Da^	20.7 ± 0.33^Cb^	20.3 ± 0.33^Bb^	11.3 ± 0.33^Dc^	17.7 ± 0.58^Bd^
50 mg/ml TCW	12.7 ± 0.33^Da^	13.3 ± 0.33^Da^	17.3 ± 0.33^Cb^	17.3 ± 0.33 ^Eb^	15.7 ± 0.58^Ce^
25 mg/ml TCM	12.3 ± 0.33^Da^	19.3 ± 0.33^Eb^	14.7 ± 0.33^Dc^	10.7 ± 0.17^Ed^	15.7 ± 0.58^Ce^
25 mg/ml TCW	9.7 ± 0.33^Ea^	10.3 ± 0.58^Fa^	14.0 ± 0.0^Db^	14.3 ± 0.44^Fb^	13.3 ± 0.58^Db^
5%DMSO	0.0^Fa^	0.0^Ga^	0.0^Ea^	0.0^Ga^	0.0^Ea^
0.2 mg/mL Nystatin					20.3 ± 0.58^A^

Mean zones of inhibition expressed as x̄ ± SD, *n* = 3. Mean values with the same uppercase (A-G) superscript letter along the columns and lowercase (a-e) superscript letter across the rows are not significantly different (*P* > 0.05; one-way ANOVA followed by Tukey’s paired test)

Methanol extract of the *T. catappa* L. exhibited greater antibacterial activity against *P. aeruginosa, E. coli, and S. aureus*, while the aqueous extract displayed greater antibacterial activity against *B. subtilis* for all tested concentrations (Table 2). The zones of inhibition of the methanol extract of the *T. catappa* L. had significant differences across all the tested microorganisms at concentrations of 25 mg/ml and 100mg/ml (*P* > 0.05 Table 2). However, methanol extracts at 50 mg/ml had a significant difference for *S. aureus, B. subtilis,* and *C. albicans* while *P. aeruginosa* and *E. coli* had no significant difference (*P* > 0.05 Table 2). The mean zones of inhibition of the aqueous extract of the *T. catappa* L. at concentrations of 25 mg/ml, 50 mg/ml, and 100 mg/ml had no significant difference between *S. aureus* and *P. aeruginosa.* Moreover*,* the aqueous extract had no significant zones of inhibition between *E. coli* and *B. subtilis* strains at all the tested concentrations (*P* > 0.05 Table 2). Comparing between the bacteria strains and the fungus revealed zones of inhibition with significant differences at all the tested concentrations for the aqueous extract (*P* > 0.05 Table 2).

The study showed a significant difference in zones of inhibition for ciprofloxacin while the negative control (5% DMSO) showed no significant difference against all the tested microorganisms (*P* > 0.05 Table 2).

**Fig. 3:**
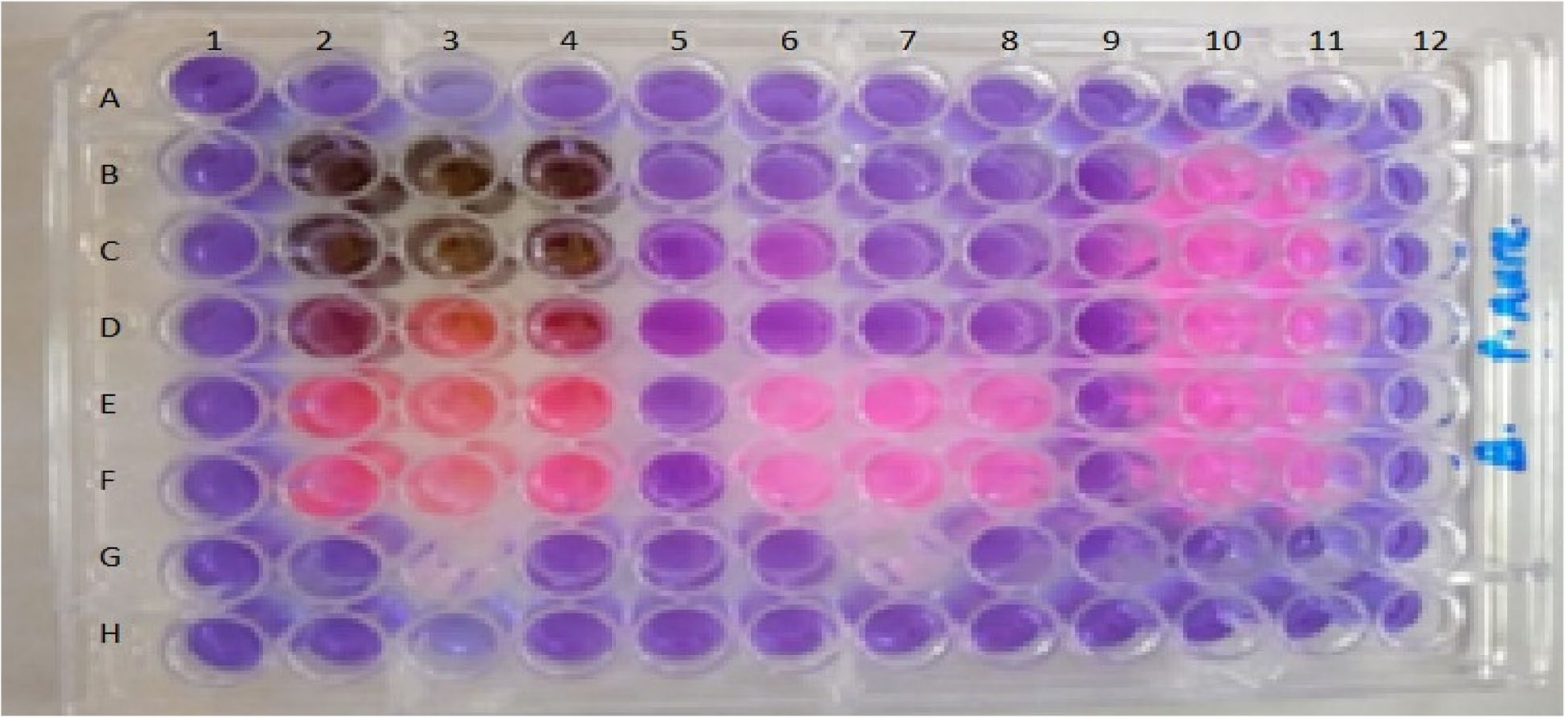
Minimum inhibition concentration of *T. catappa* L. extract, ciprofloxacin, and 5% DMSO against *P. aeruginosa*. Wells 2–4 from rows B-F contained two-fold dilution of plant’s extract, wells 6–8, rows B-F contained two-fold dilution of ciprofloxacin, wells 10–11, rows B-F contained 5% DMSO, while wells 2–11 in row G contained abiotic control (media, and resazurin dye) and all the wells in columns 1, 5 9 and 12, and rows A and H contained sterile distilled water and resazurin dye

The summary of the lowest concentration (MIC) of the *T.catappa* L. extracts that prevented the visible growth of microorganisms is depicted (Fig. 3 and Table 3). The MIC values ranged from 1563 to 12,500 µg/ml for the crude extracts whereas that of the standard solutions ranged from 5 to 0.03125 μg/ml as depicted in Table 3. The bactericidal and bacteriostatic impact of the *T. catappa* L. extracts were evaluated utilizing the proportion MBC/MIC [33].

**Table 3:** Table showing the MIC, MBC, and MFC of the *T. catappa* L. methanol and aqueous extract in μg/ml

**Microorganism**		**MIC (**μg/ml)	**MBC (**μg/ml)	**MBC/MIC (**μg/ml)	**Activity**
***S. aureus***	Ciprofloxacin	0.25	-	-	Bacteriostatic
	TCM	6,250	6,250	1	Bactericidal
	TCW	12,500	12,500	1	Bactericidal
***P. aeruginosa***	Ciprofloxacin	0.125	0.5	4	Bactericidal
	TCM	6,250	6,250	1	Bactericidal
	TCW	6,250	6,250	1	Bactericidal
***E. coli***	Ciprofloxacin	0.03125	-	-	Bacteriostatic
	TCM	6,250	-	-	Bacteriostatic
	TCW	3,125	6,250	2	Bactericidal
***B. subtillis***	Ciprofloxacin	0.5	-	-	Bacteriostatic
	TCM	6,250	6,250	1	Bactericidal
	TCW	12,500	12,500	1	Bactericidal
		**MIC**	**MFC**	**MFC/MIC**	
***C. albicans***	Nystatin	5	-	-	Fungistatic
	TCM	1,563	-	-	Fungistatic
	TCW	1,563	-	-	Fungistatic

The minimum inhibition concentration, minimum bactericidal concentration, and minimum fungicidal concentration of *T. catappa L.* methanol (TCM) and aqueous extracts

### Antioxidant assay

#### Evaluation of free radical scavenging activity using DPPH

The capacity of the crude extracts to reduce the stable free radical from DPPH was used as a quantitative determination of free radical scavenging properties [29]. This was determined by measuring absorbance using a UV–vis spectrophotometer at a wavelength of 517 nm [28]. The relative scavenging activities and IC_50_ values of the extracts and ascorbic acid were determined (Table 4).

**Table 4:** Table showing % relative scavenging activity (RSA) and IC_50_ values of L-Ascorbic acid, the T. catappa L. methanol, and aqueous extracts

% Relative scavenging activity (RSA)			
**Concentration (μg/ml)**	**L-Ascorbic acid**	***T. catappa* L. methanol**	***T. catappa* aqueous extract**
	**Mean ± SD**	**Mean ± SD**	**Mean ± SD**
**1000**	91.97 ± 0.11^Aa^	82.58 ± 0.17^Ab^	81.70 ± 0.39^Ac^
**200**	91.35 ± 0.39^Aa^	81.85 ± 0.68^Ab^	80.41 ± 0.75^Ab^
**40**	90.01 ± 0.34^Ba^	80.96 ± 0.39^Bb^	75.17 ± 0.37^Bc^
**8**	44.33 ± 0.12^Ca^	38.84 ± 0.70^Cb^	39.43 ± 0.60^Cb^
**1.6**	13.24 ± 0.31^Da^	7.36 ± 0.24^Db^	14.28 ± 0.28^Dc^
**0.32**	10.81 ± 0.32^E^	3.42 ± 0.34^E^	7.42 ± 0.39^E^
**0.0**	0.00 ± 0	0.00 ± 0	0.00 ± 0
***IC***_**50**_	**8.723**	**13.04**	**13.42**

%RSA values are expressed as x̄ ± SD, *n* = 3. Values with the same uppercase (A-E) superscript letter along the columns and lowercase (a-d) superscript letter across the rows are not significantly different (*P* > 0.05; one-way ANOVA followed by Tukey’s paired test)

The results showed a significant difference in % radical scavenging activity at concentrations of 0.32 μg/ml, 1.6 μg/ml, 8 μg/ml, and 40 μg/ml for L-ascorbic acid, the *T.catappa* L. methanol and aqueous extracts (*P* > 0.05 Table 4). At 200 μg/ml and 1000 μg/ml, there was no significant difference (*P* > 0.05) for L-ascorbic, and the *T.catappa* L. methanol and aqueous extracts.

At all the tested concentrations there was a significant difference in the percentage radical scavenging activity of the L-ascorbic acid relative to the *T.catappa* L. extracts (P > 0.05 Table 4). The study revealed significant difference in the percentage radical scavenging activities of the *T.catappa* L. methanol and aqueous extracts at 1000 μg/ml, 40 μg/ml, 1.6 μg/ml, and 0.32 μg/ml at 40 while there was no significant difference at 200 μg/ml and 8 μg/ml (*P* > 0.05 Table 4). The study further determined the concentration of the extracts that were able to scavenge the DPPH-free at 50% (IC_50_). The methanol and aqueous extracts of the *T.catappa* L. had an IC_50_ value of 13.04 μg/ml and 13.42 μg/ml while the L-ascorbic acid had an IC_50_ value of 8.723 μg/ml (Table 4).

#### Determination of potassium ferricyanide reducing antioxidant power (pFRAP)

The ferric reducing properties of the *T. catappa* L. extracts was determined using potassium ferri-cyanide which was reduced to potassium ferro-cyanide. The reducing power was determined by measuring the absorbance values at 700 nm, and the results are as tabulated (Table 5).

**Table 5:** Table showing Ferric reducing antioxidant power (FRAP) of T. catappa L. aqueous and methanolic extracts

Concentration (μg/ml)	Ferric reducing power		
	**L-Ascorbic acid**	***T. catappa* L. methanol extract**	***T. catappa* L. aqueous extract**
	**Mean** ± **SD**	**Mean** ± **SD**	**Mean** ± **SD**
**1000**	0.425 ± 0.004^Aa^	0.587 ± 0.003^Ab^	0.453 ± 0.003^Ac^
**200**	0.418 ± 0.001^Ba^	0.513 ± 0.003^Bb^	0.438 ± 0.002^Bc^
**40**	0.412 ± 0.002^Ba^	0.503 ± 0.002^Cb^	0.430 ± 0.004^Bc^
**8**	0.403 ± 0.002^Ca^	0.482 ± 0.005^Db^	0.417 ± 0.002^Cc^
**1.6**	0.394 ± 0.001^Da^	0.463 ± 0.006^Eb^	0.409 ± 0.003^Cc^
**0.32**	0.370 ± 0.003^Ea^	0.437 ± 0.001^Fb^	0.397 ± 0.008^Dc^

Mean absorbance values are expressed as x̄ ± SD, *n* = 3. Values with the same uppercase superscript letter along the columns and lowercase superscript letter across the rows are not significantly different (*P* > 0.05; one-way ANOVA followed by Tukey’s paired test)

The absorbance values for L-ascorbic acid recorded at concentrations of 0.32 μg/ml, 1.6 μg/ml, 8 μg/ml, and 1000 μg/ml had a significant difference while at 40 μg/ml and 200 μg/ml, there was no significant difference (*P* > 0.05 Table 5). The mean absorbance values for the methanol extract of the *T. catappa* L. at all the tested concentrations were significantly different (*P* > 0.05 Table 5). The *T. catappa* L. aqueous extracts absorbance values at 1.6 μg/ml and 8 μg/ml, and 40 μg/ml and 200 μg/ml were not significantly different while at 0.32 μg/ml and 1000 μg/ml, the values were significantly different (*P* > 0.05 Table 5).

Moreover, the comparison among the L-ascorbic acid and the *T. catappa* L. methanol and aqueous extracts was done. The study revealed a significant difference in the absorbance values at all the tested concentrations for the standard and the extracts (*P* > 0.05 Table 5).

## Discussion

In recent years there has been an increase in drug resistance, which poses a huge threat to public health across the world. Majority of the available antibiotics have been compromised by the multidrug resistant microbes [7]. Additionally non-communicable diseases such as diabetes, cancer and neurodegenerative diseases are on the rise. The main etiology is oxidative stress which arise due to higher free radicals than the antioxidants. The drugs used are costly, only alleviate symptoms and are associated with adverse effects [34]. Natural products have become an alternative source of medicine that are extensively used in management of many disease including the microbial infections and oxidative related diseases [35]. These products are characterized with numerous phytochemicals and exhibit a wide range of biological activities including antimicrobial, antioxidant etc. The increased preference of these products is as result of their high efficacy, less or no toxic effects and lower cost of acquisition [2].

The study found that methanol and aqueous extracts of the *T. catappa* L. contained alkaloids, tannins, steroids, cardiac glycosides, flavonoids, phenols, saponins, and coumarins, while terpenoids was absent. These phytochemicals are responsible for the antimicrobial and antioxidants properties as reported by various authors [9, 22]. These findings correlated with those in the study of Muhammad et al. [32] that reported the presence of tannins, alkaloids, steroids, and saponins in the ethanol extract of *T. catappa* L. The detected phytochemicals are reported to exhibit different pharmacological activities with antimicrobial and antioxidant properties [36]. A study by Ruto et al. [9], demonstrated antimicrobial and antioxidant properties in methanolic crude extracts of *Entada leptostachya* and *Prosopis juliflora* and this could be due to phytochemicals flavonoids, terpenoids, phenols, and saponins that had been reported. Also, Madivoli et al. [22] reported phenolic compounds such as tannins, in ethyl acetate and methanol extracts of the *Prunus africana* and *Harrisonia abyssinica* as being linked to antimicrobial and antioxidant properties. Phytochemicals exhibit antimicrobial activity via different mechanisms. For instance, flavonoids have been reported to have antimicrobial activity due to their ability to complex with extracellular cells and soluble proteins while tannins have the ability to inactivate microbial adhesions [37].

The determination of functional groups by FT-IR revealed the presence of various functional groups such as OH, C = C, C-O, and C–C. These functional groups are linked to the presence of phytochemical compounds present in the extracts. Similar functional groups were reported by Agu et al. [38] on the *Terminalia catappa L. kernel* oil using response surface methodology and artificial neural network.

The presence of C = O, C = C, and CH_2_ have been reported in Seaweed *Dictyota dichotoma* by Imran et al. [39]*.* In another study Purnama et al. [40] reported the bands such as O–H, C-H, N–H, and C = O present in the extract of *Lantana camara* L. were linked to metabolites such as amides, esters, phenolic, and aldehydes.

The methanol extract of *T. catappa* L. demonstrated high antimicrobial activity against the studied microbes. The antimicrobial activity was higher against gram-negative bacteria strains as compared to gram positive bacteria. However these findings contradicted those of Manzur et al*.* [41] study which reported higher antimicrobial of the acetone, methanol, and N, N- dimethylformamide extracts of the *T. catappa* L. extracts against gram positive bacteria. Similarly, the methanol and aqueous extracts of *T. catappa* L demonstrated antifungal activity against *C. albicans*. The extracts exhibited higher antifungal activity at higher concentration that was comparable to nystatin, the antifungal agent. The zones of inhibition of the extracts and positive controls varied depending on the concentrations [10]. These findings are in line with those reported by Tercas et al. [42], on the study of the phytochemical and antifungal activity of *T. catappa* L bark extracts. The study found out that, methanol and aqueous extracts of the *T. catappa* L. had bactericidal effects on most of the microorganisms. Similar results were demonstrated bpery Mbengui et al. [33] on alcoholic and aqueous extracts of *T. catappa* extracts. In the study alcoholic extract had better zones of inhibition ranging from 8.7–15.4 mm while that of aqueous was between 0–10 mm [33]. The antimicrobial properties of herbal plants are attributed to the existence of secondary metabolites such as flavonoids, tannins, steroids, saponins, and diterpenoids [32]. Constituents such as alkaloids are known to interfere with cell division of the bacteria by inter-chelating with DNA of both Gram-negative and Gram- positive bacteria while steroids results in leakage of membrane as a result of the formation of complexes with the membrane lipids [10]. Phenolics compounds are antimicrobial agents that are known to inhibit biofilm formation, synthesis of nucleic acid and reduction of host adhesion and neutralization of bacterial toxins [43].

The MIC values for methanol extract had a lower or the same value than that of aqueous extract for all the tested bacteria strains except for *Escherichia coli*. This showed that methanol extract has better activity than aqueous extract of the *T. catappa* L. The aqueous extract may contain lower antimicrobial constituents which is explained by the fact that most decoctions are taken in large quantities over a long period of time [10]. This MIC values reported in this study are similar to those reported by Ihuma et al. [44], who reported MIC values of 3.125 mg/ml on the *T. catappa* extracts.

The concentration of the test solutions that scavenged 50% of DPPH (IC_50_) was found to be 8.723 µg/ml, 13.42 µg/ml, and 13.04 µg/ml for L-ascorbic acid, the *T. catappa* L. methanol and aqueous extracts respectively (Table 4). The relative scavenging activity of both L-ascorbic acid and the *T. catappa* L. extracts increased with an increase in concentration. Annegowda et al. [45] repported similar results on hyrolysed extracts of *T. catappa* L. leaf. The power to reduce ferric to ferrous complex by extracts is an indication of the ability to donate electrons which is an approach for analyzing the antioxidant properties of the extracts. The findings of this study were in agreement with previous studies carried out by Poongulali et al. [46] on the methanol extract of *T. catappa* extract, which demonstrated concentration dependent ferric reducing power. Phenolic compounds are responsible for the antioxidant activity due to the presence of one or more hydroxyl groups in their structure that are able to scavenge the free radicals [47]. Therefore the antioxidant activity of *T. catappa* L. extracts can be attributed to the presence of phenolics. Phenolics acts as an antioxidant agent by inhibiting lipid peroxidation and exhibiting physiological activities [43].

## Conclusion

Based on the obtained results, the methanol and aqueous extracts of the *T. catappa* L. have remarkable antioxidant and antimicrobial activities. The extracts were able to scavenge free radicals from DPPH and also reduce ferric ions. The extracts showed remarkable antimicrobial properties against four bacterial and fungus strains. Further studies are required isolate and characterize the specific compounds responsible for the antioxidant and antimicrobial activities and also to determine the *in-vivo* activities of the compounds in the *T. catappa* L. extracts.

### Supplementary Information

**Supplementary Material 1.**

## Data Availability

All data generated or analysed during this study are included in this published article and its Supplementary files.
