# Supplementary material for: Phytochemical characterization, antimicrobial and antioxidant activities of Terminalia catappa methanol and aqueous extracts
Source: BMC Complement Med Ther. 2024 Apr 2;24:137. doi: 10.1186/s12906-024-04449-7 (PMC10986026; doi:10.1186/s12906-024-04449-7)
Supplement: Supplementary file 1 — Supplementary Material 1. [file 12906_2024_4449_MOESM1_ESM.pdf]

Supplementary Table 1: Table showing the average diameter of zones of inhibition of *T. catappa* L. methanol extract (TCM) against five microorganisms

	Mean ± SD			
**Sample**	***S. aureus***	***P. aeruginosa***	***E. coli***	***B. subtilis***
**0.15 mg/ml ciprofloxacin**	21.0 ±0 ^{Aa}	27.7 ±0.33^{Ab}	25.7 ±0.17 ^{Ac}	26.3 ±0.33 ^{Ad}
**100 mg/ml TCM**	18.5 ±0.29^{Ba}	25.7 ±0.33^{Bb}	25.0 ±0.0 ^{Ab}	14.3 ±0.33 ^{Bc}
**50 mg/ml TCM**	13.7 ±0.58^{Ca}	20.7 ±0.33^{Cb}	20.3 ±0.33 ^{Bb}	11.3 ±0.33 ^{Cc}
**25 mg/ml TCM**	12.3 ±0.33 ^{Da}	19.3 ±0.33^{Db}	14.7 ±0.33 ^{Cc}	10.7 ±0.17 ^{Dd}
**5%DMSO**	0.0^{Ea}	0.0^{Ea}	0.0 ^{Da}	0.0 ^{Ea}

[1]

Supplementary Table 2: Table showing the average diameter of zones of inhibition of T. catappa L. aqueous extract (TCW) against five microorganisms

	Mean ± SD			
**Sample**	***S. aureus***	***P. aeruginosa***	***E. coli***	***B. subtilis***
**0.15 mg/ml ciprofloxacin**	21.0 ±0 ^{Aa}	27.7 ±0.33^{Ab}	25.7 ±0.17 ^{Ac}	26.3 ±0.33 ^{Ad}
**100 mg/ml TCW**	15.3 ±0.33^{Ba}	15.7 ±0.33^{Ba}	20.3 ±0.33 ^{Bb}	19.3 ±0.33 ^{Bb}
**50 mg/ml TCW**	12.7 ±0.33^{Ca}	13.3 ±0.33^{Ca}	17.3 ±0.33 ^{Cb}	17.3 ±0.33 ^{Cb}
**25 mg/ml TCW**	9.7 ±0.33 ^{Da}	10.3 ±0.58^{Da}	14.0 ±0.0 ^{Db}	14.3 ±0.44 ^{Db}
**5%DMSO**	0.0^{Ea}	0.0^{Ea}	0.0 ^{Ea}	0.0 ^{Ea}

[2]

[1] Mean zones of inhibition expressed as $\bar{x}$ ± SD, n=3. Mean values with the same uppercase superscript letter along the columns and lowercase superscript letter across the rows are not significantly different (P>0.05; one-way ANOVA followed by Tukey's paired test).

[2] Mean zones of inhibition expressed as $\bar{x}$ ± SD, n=3. Mean values with the same uppercase superscript letter along the columns and lowercase superscript letter across the rows are not significantly different (P>0.05; one-way ANOVA followed by Tukey's paired test).
